# Severe aortic valve regurgitation in patient with Takayasu arteritis: a case report

**DOI:** 10.1093/ehjcr/ytae473

**Published:** 2024-09-04

**Authors:** Vasiliki Tassi, Dimitrios Tzalas, Elektra Papadopoulou, Athanasios Trikas

**Affiliations:** Department of Cardiology, Evangelismos General Hospital, Υpsilantou Str. 45-47, 10676 Athens, Greece; Department of Cardiology, Evangelismos General Hospital, Υpsilantou Str. 45-47, 10676 Athens, Greece; Department of Cardiology, Evangelismos General Hospital, Υpsilantou Str. 45-47, 10676 Athens, Greece; Department of Cardiology, Evangelismos General Hospital, Υpsilantou Str. 45-47, 10676 Athens, Greece

**Keywords:** Large vessel vasculitis, Inflammatory arteritis, Vascular stenosis, Angiography, Thickening of aortic wall, Aortic regurgitation, Case report

## Abstract

**Background:**

Takayasu arteritis (TAK) is a systemic non-inflammatory vasculitis that primarily affects large- and medium-sized arteries.

**Case summary:**

We report the case of a 57-year-old woman with a history of coronary artery bypass grafting (CABG) 7 years prior, who was referred for a stress echo due to chest pain. Transthoracic echocardiography revealed the left ventricle at the upper limits of normal with preserved contractility, as well as circumferential thickening of the aortic root, causing severe aortic regurgitation (AR). Cardiac computed tomography and angiography demonstrated diffuse thickening of the aortic wall from the aortic root to the descending thoracic aorta, extending to the left carotid artery and significant stenosis of the left subclavian artery. Coronary angiography showed severe narrowing of the left main coronary ostium with ostial stenosis and total occlusion of the right coronary and left internal mammary arteries. Magnetic angiography highlighted thickening of the aortic wall, while no active inflammation was detected on positron emission tomography. These findings suggested Takayasu aortitis with chronic inflammation.

**Discussion:**

In young patients, particularly women, who present with angina and coronary ostial stenosis, Takayasu arteritis should be considered in the differential diagnosis. Aortic regurgitation (AR) is a serious complication, and its surgical management can be challenging.

**Learning points**

- To identify the key clinical features that suggest a diagnosis of TAK
- To understand the role of imaging studies, particularly advanced vascular imaging techniques, such as MRI, CT angiography, and PET scan, in confirming the diagnosis of TAK by visualizing characteristic arterial lesions.
- To emphasize the importance of tailoring therapy based on disease stage and extent of vascular involvement.

VT conceived the idea for the case report presentation and took the lead in writing the manuscript, with significant support and contributions from DT and EP. VT was responsible for conducting the literature review, ensuring a comprehensive analysis of relevant sources. EP and AT also took charge of patient follow-up and managed the data collection process, ensuring accuracy and consistency. All authors participated in reviewing the results and have approved the final version of the manuscript.

## Introduction

Takayasu arteritis (TAK) is a chronic idiopathic granulomatous large-vessel vasculitis that affects the aorta and its primary branches, with an incidence of 1 to 3 per million. In 7% to 9% of cases, the disease involves the coronary arteries. Patients with TAK may present with non-specific symptoms that are often attributed to other causes or go unrecognized for several months or years prior to diagnosis.^1^ The chronic nature of the disease and the requirement for long-term immunosuppressive therapy pose challenges in lifelong surveillance and quality of life. Aortic regurgitation is a serious complication of TAK associated with high mortality, and its surgical management is particularly challenging. Herein, we report a female patient with Takayasu arteritis, coronary artery involvement, and severe aortic regurgitation.^1–4^

## Summary figure

**Table:** 

Category	Details
**Patient History**	
Exertional Chest Pain	Present over the last 6 months
Blood Pressure Difference	Inter-arm blood pressure difference greater than 10 mm Hg
Pulse Examination	Decreased brachial artery pulse in the left arm
Limb Symptoms	Upper left limb claudication
Heart Murmur	Diastolic murmur noted at the left third intercostal space
**Imaging Findings**	
Echocardiography	Severe aortic regurgitation, thickening of the aortic wall
CT Angiography	Excluded aortic dissection, confirmed thickening of the aortic wall from the aortic root to the descending thoracic aorta and significant stenosis of the left subclavian artery
Coronary Angiography	Confirmed ostial stenosis of left main artery and three-vessel disease
Magnetic Resonance Imaging	Confirmed thickening of the aortic wall. No increase in T1 and T2 signals to suggest aortic wall edema
Pet Scan	No Active Inflammation, No Aortic Wall Edema

## Case presentation

A 57-year-old woman was referred to our centre for stress echocardiography due to exertional chest pain over the last 6 months. Her past medical history included coronary artery bypass grafting (CABG) performed 7 years ago. Physical examination revealed an inter-arm blood pressure difference greater than 10 mm Hg, a decreased brachial artery pulse in the left arm, and upper left limb claudication. A diastolic murmur was noted at the left third intercostal space. She was afebrile, with stable vital signs and decreased diastolic pressure. Her medication at the time of the echocardiography included aspirin 100 mg QD and atorvastatin 40 mg QD.

**Figure 1:**
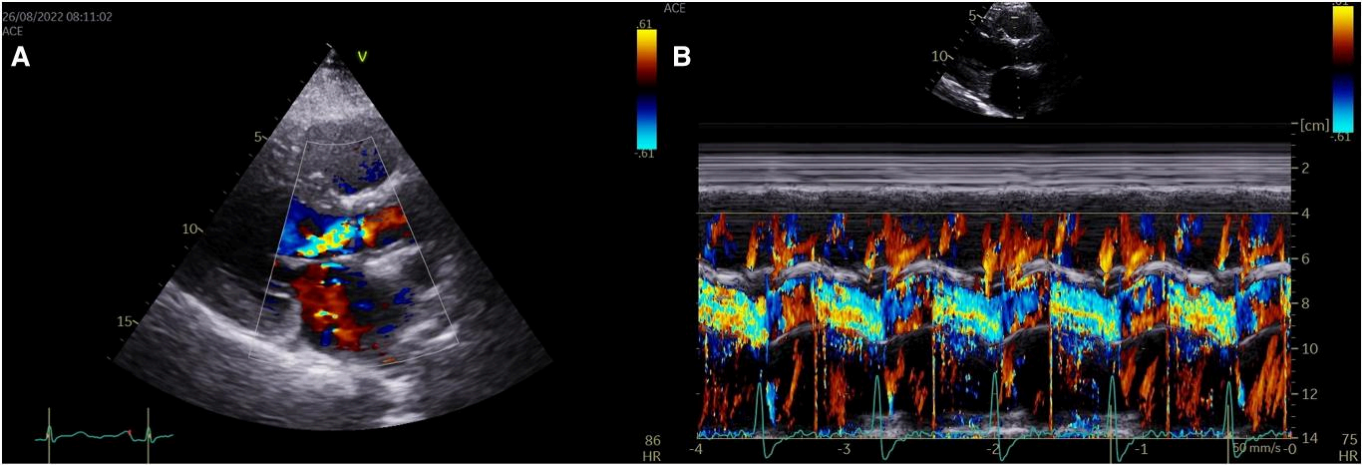
(*A*) Transthoracic echocardiography, parasternal long-axis view, showing severe aortic regurgitation. *(B*) M-mode with colour in the parasternal long-axis view, also showing severe aortic regurgitation.

**Figure 2:**
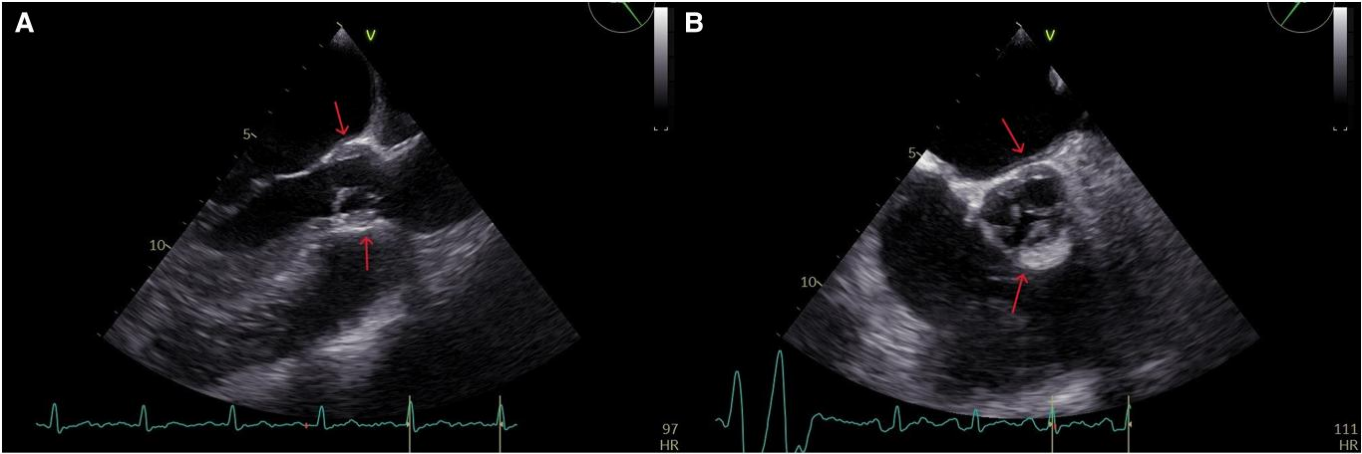
Transesophageal echocardiography in both the three-chamber (*A*) and short-axis views (*B*), showing circular thickening of the aortic root (indicated by the red arrow).

Transthoracic echocardiography revealed a left ventricle with a left diastolic diameter at the upper limit of normal, an ejection fraction (EF) of approximately 50%, and thickening of the aortic root in a circular manner, causing severe aortic regurgitation (AR), as seen in *Figure 1* and Video 1 in the Supplementary material online. Transesophageal echocardiography (TOE) confirmed these findings, as seen in *Figure 2* and Video 2 in the Supplementary material online. We immediately proceeded with computed tomography angiography (CTA) to exclude aortic dissection and intramural hematoma of the aorta. The patient was admitted to the Cardiology ward to investigate the aetiology of AR.

Her electrocardiogram showed no abnormalities, while routine hematologic and biochemical investigations, including erythrocyte sedimentation rate (ESR), C-reactive protein (CRP), and multiple blood cultures, were normal.

**Figure 3:**
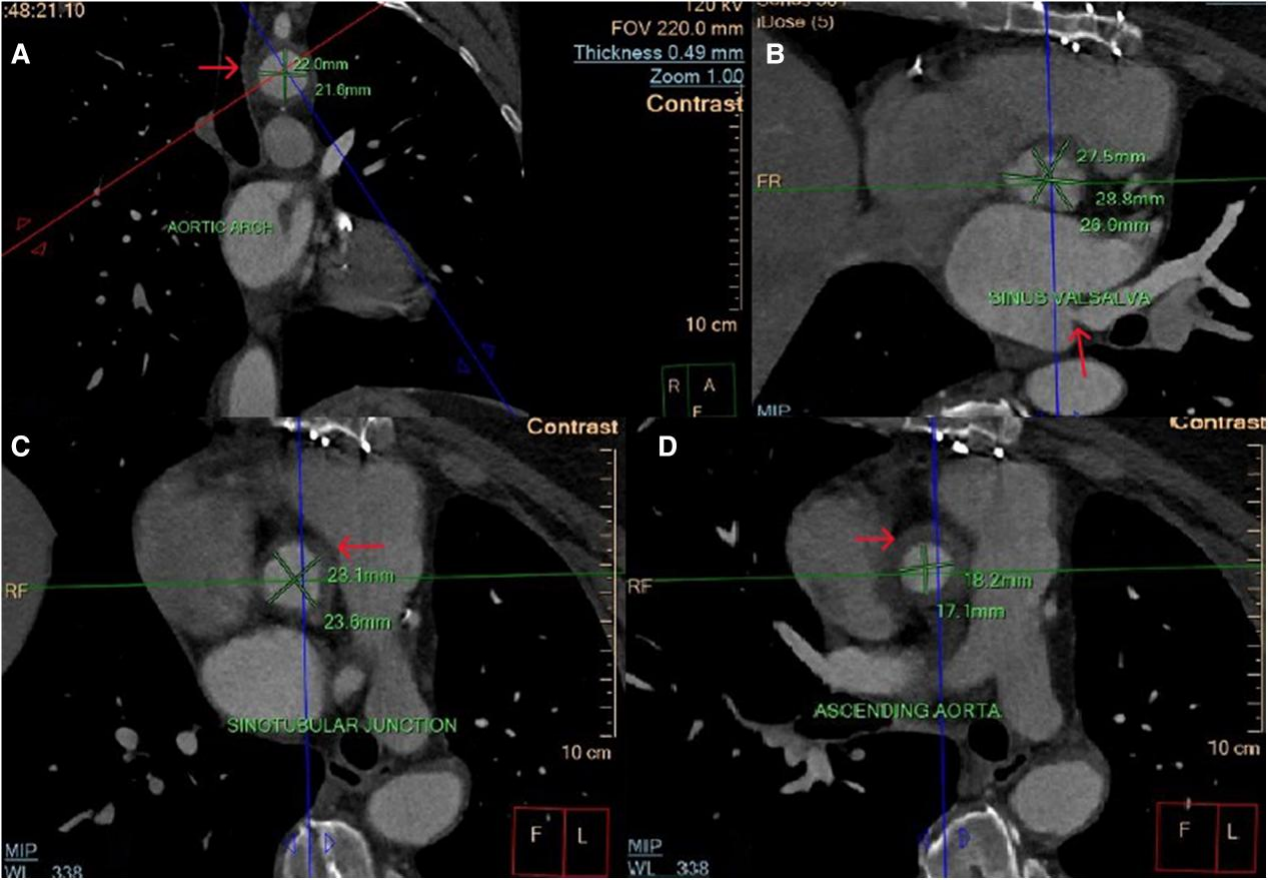
Cardiac computed tomography and angiography reveal circumferential thickening of the aortic wall extending from the aortic root to the descending thoracic aorta. Series of CT images (from *A* to *D*) presented here illustrates the progressive thickening of the aortic wall. These post-contrast images are captured in axial view. The red arrow in image B shows the ostial stenosis of the left main artery.

**Figure 4:**
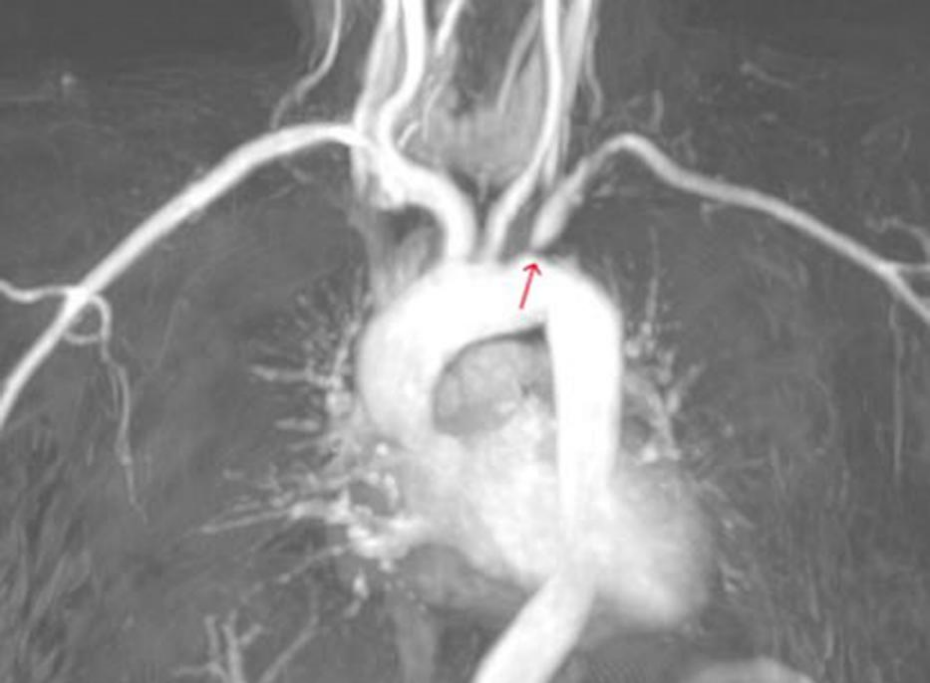
Contrast-enhanced three-dimensional magnetic resonance angiography reveals the aortic arch and great vessels, demonstrating significant narrowing of subclavian artery (indicated by the red arrow).

**Figure 5:**
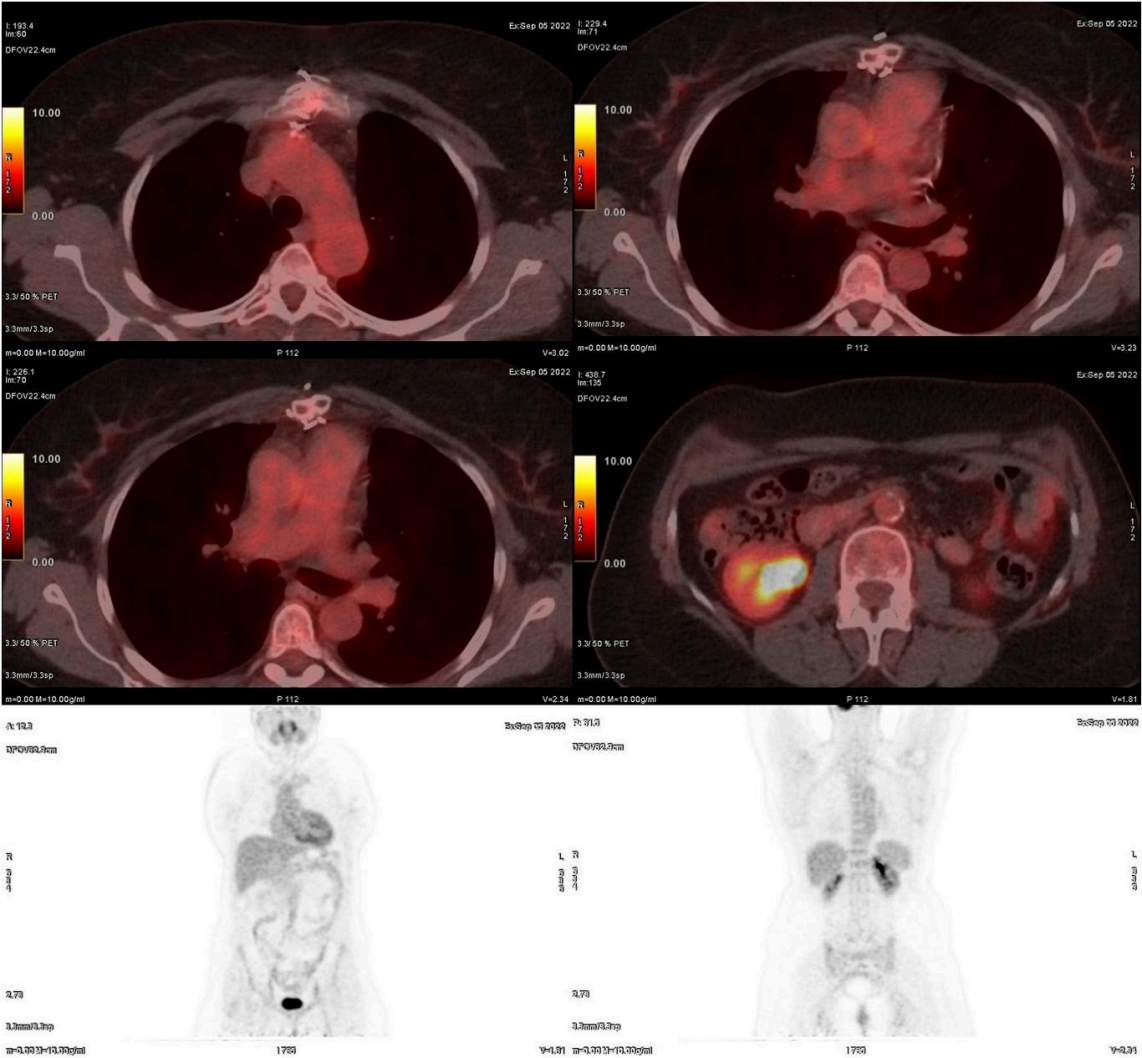
Representative 18F-FDG PET scan showing no evidence of active inflammation. The scan reveals normal physiological uptake of 18F-FDG, with no abnormal areas of increased tracer accumulation typically indicative of inflammatory activity. The absence of hypermetabolic regions suggests that the patient does not have active inflammatory processes at the time of imaging.

Cardiac computed tomography and angiography revealed circumferential thickening of the aortic wall at the level of the aortic root to the descending thoracic aorta, with extension to the left carotid artery, brachiocephalic artery, and left subclavian artery, causing significant stenosis, as seen in *Figure 3*. Coronary angiography demonstrated triple vessel disease with severe ostial narrowing of the left main coronary artery, total occlusion of the right coronary artery (RCA) and left mammary artery, and occluded saphenous vein graft to RCA (Video 3 in the Supplementary material). Contrast-enhanced three-dimensional magnetic resonance angiography (MRA) was subsequently performed, providing excellent assessment of the vascular wall and lumen. The MRA highlighted the thickened aortic wall from the aortic root to the descending thoracic aorta, with abundant fibrous elements within it. No increase in T1 and T2 signals to suggest aortic wall edema (*Figure 4*) was noted. On Day 15, positron emission tomography with 2-deoxy-2-[fluorine-18] fluoro-D-glucose integrated with computed tomography (18F-FDG PET/CT) was performed, and no active inflammation was detected (*Figure 5*). Severe aortic regurgitation and wall thickening of the aortic annulus, thoracic aorta, and its branches as revealed initially from echocardiography and confirmed with CTA and MRA, the characteristic pattern of Type 1 coronary lesions (occlusion of the ostial segments) on coronary angiography and lack of active inflammation on 18F-FDG PET scan suggested Takayasu aortitis, not in an active phase (stage IIB), according to the Modified Ishikawa Criteria (see Supplementary material online). The patient was treated with methotrexate and oral glucocorticoids. Despite the recommendation for surgical intervention of coronary artery disease and aortic regurgitation, the patient declined the procedure, citing the complexities and risks associated with it.

## Discussion

The diagnosis of Takayasu arteritis is supported by the presence of symptoms that indicate involvement of the aorta and its main branches.^1^ In this case, the discrepancy in blood pressure readings between the arms was an important clinical marker of vascular involvement due to stenosis of the subclavian artery. Upper limb claudication was a direct effect of the disease causing artery stenosis and an indication of ischaemia caused by reduced blood flow. Contrast enhanced cardiac computed tomography had provided critical imaging evidence, revealing thickening of the aortic wall and representing the inflammatory process which affects the vessel walls. This inflammation can lead to stenosis, occlusion, or aneurysmal dilation over time. We conducted a contrast-enhanced MRA to further evaluate the vascular involvement seen on CT. Therefore, the combination of clinical signs and advanced imaging findings strongly supports the diagnosis of Takayasu arteritis in this patient.^5,6^

The initial step in managing TAK involves controlling the underlying inflammation with immunosuppressive therapy. Corticosteroids remain the cornerstone of treatment, often combined with other immunosuppressive agents, such as methotrexate or azathioprine. Following rheumatology evaluation and according to the ESC guidelines on aortic disease, the patient was started on methotrexate and oral corticosteroids due to extensive target organ damage. Rheumatologists tapered steroids after 1 month of initiation and used methotrexate, a non-glucocorticoid immunosuppressive agent, as first-line treatment, according to the 2021 Guidelines of the American College of Rheumatology.^5,6^ They recommended continuing the current immunosuppressive therapy, as there was no evidence of inflammation or progression of previously identified vascular lesions seen on imaging under close follow-up.^5^ In our case, cardiac surgery was recommended for severe aortic regurgitation (AR) and coronary artery disease, but the patient declined surgical management due to the high reported perioperative mortality risk, which was attributed to the presence of extensive fibrosis and tissue fragility. In the context of surgery, these fragile and fibrotic tissues present significant challenges. The inflamed vessel walls are prone to tearing, which can lead to severe complications during or after the procedure. Moreover, the healing process in these patients can be impaired due to the ongoing inflammation, increasing the risk of post-surgical complications, such as infections, poor wound healing, or even further damage to the arteries. In this case, the decision-making process was thorough and involved a multidisciplinary team, including rheumatologists, vascular surgeons, and interventional radiologists.

Given these risks, our patient perceived surgery as a high-risk option with uncertain outcomes. She was concerned that the potential benefits of correcting aortic regurgitation might not outweigh the risks of surgery, including the possibility of worsening their condition or facing life-threatening complications. This concern, combined with the knowledge of condition's complexity, led her to opt for non-surgical management strategies, despite the severity of symptoms.

Due to the relative rarity of the disease, no standardized guidelines exist for the surgical management of TAK or the best surgical approach in this population. It is unclear whether isolated aortic valve replacement (AVR) or combined aortic valve and root replacement (CAVRR) should be performed.^7,8^ A review of 27 studies encompassing a total of 194 cases (77% female) focused on the surgical management of AR in TAK. Significant causes of postoperative morbidity, mortality, and reoperation after surgical management of AR in TAK, even in the context of anti-inflammatory therapy, include prosthetic valve or graft dehiscence, pseudoaneurysm formation, periprosthetic leaks, detachment of prosthetic valves, and aneurysm formation at the anastomoses.^9^ Outcomes seem to improve in patients who receive adequate immunosuppression to achieve disease remission prior to surgery.^9^

In most centres, coronary artery bypass grafting (CABG) remains the preferred option for patients with symptomatic coronary disease despite medical therapy. Alternative interventions include angioplasty (PCI) with or without stent insertion and coronary endarterectomy.^9,10^ A major issue in patients with TAK undergoing CABG is the restenosis of the grafts. Rates of restenosis are higher following PCI compared to CABG at follow-up of 100 months (63% vs. 25%).^11^ The optimal method of revascularization is yet to be determined due to the small number of cases, lack of comparative studies, short-term follow-up, and no data comparing the two methods. In one study, 31 patients with TAK and coronary artery involvement who underwent either PCI or CABG were analysed. The study showed that CABG treatment appeared to increase long-term survival for TAK patients with coronary artery involvement.^12,13^ Another study analysed one centre's experience from 942 patients who underwent percutaneous intervention strategies (PCI) to treat 2450 arterial lesions. They concluded that most vascular lesions in TAK can be effectively and durably treated using predominantly stent-based PCI.^14^

At 2-year follow-up, no significant changes in symptomatic status or echocardiographic findings were noted.

In summary, Takayasu arteritis should be considered in the differential diagnosis, especially in young patients, particularly women, who present with angina and coronary ostial stenosis.^1–4^ Early diagnosis, effective therapy, and continued surveillance are essential for successful long-term outcomes. Long-term immunosuppressive therapy may be required to induce disease remission, although treatment success is questionable and disease relapse is not uncommon. Decision between open surgery and endovascular approaches may depend on local availability, expertise, and patient preferences.

## Supplementary Material

ytae473_Supplementary_Data

## Data Availability

All data are incorporated into the article and its online Supplementary material. The data underlying this article are available in the article and in its online Supplementary material.

## Lead author biography

Vasiliki Tassi is currently a cardiology resident at Evangelismos Hospital, Athens, Greece. Vasiliki Tassi completed her Master Degree on Interventional Cardiology at National and Kapodistrian University of Athens-Ippokrateion Hospital in May 2023. Her scientific interest is in cardiac imaging and echocardiography.

## Supplementary material

**Consent:** The authors confirm that written informed consent for submission and publication of this case report, including images, and associated text has been obtained from the patient in line with COPE guidance.
