# Supplementary figures and images for: Severe aortic valve regurgitation in patient with Takayasu arteritis: a case report
Source: Eur Heart J Case Rep. 2024 Sep 4;8(9):ytae473. doi: 10.1093/ehjcr/ytae473 (PMC11420678; doi:10.1093/ehjcr/ytae473)

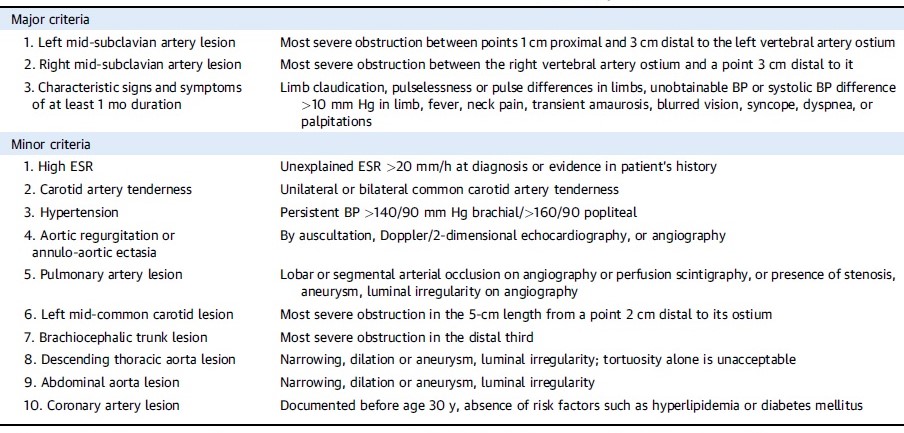

Supplement: ytae473_Supplementary_Data [file ytae473_supplementary_data.zip › Criteria .jpg]
